# Homogenous–heterogeneous reactions in MHD flow of Powell–Eyring fluid over a stretching sheet with Newtonian heating

**DOI:** 10.1007/s00521-017-2943-6

**Published:** 2017-03-24

**Authors:** Imad Khan, M. Y. Malik, T. Salahuddin, Mair Khan, Khalil Ur Rehman

**Affiliations:** 1 Department of Mathematics, Quaid-i-Azam University, Islamabad, 44000 Pakistan; 2 Mirpur University of Science and Technology (MUST), Mirpur, Pakistan

**Keywords:** Powell–Eyring fluid model, Homogenous–heterogeneous reactions, Stretching sheet, Newtonian heating

## Abstract

This article addresses the effects of homogenous–heterogeneous reactions on electrically conducting boundary layer fluid flow and heat transfer characteristics over a stretching sheet with Newtonian heating are examined. Using similarity transformations, the governing equations are transformed into nonlinear ordinary differential equations. The constricted ordinary differential equations are solved computationally by shooting technique. The impact of pertinent physical parameters on the velocity, concentration and temperature profiles is discussed and explored via figures and tables. It is clear from figures that the velocity profile reduces for large values of fluid parameter *B* and Hartmann number *H*. Skin friction coefficient decreases for large values of Hartmann number *H* and fluid parameter *B*. Also, heat transfer rate monotonically enhances with conjugate parameter of Newtonian heating *γ* and Prandtl number *Pr*.

## Introduction

 Fluids used in industries maximum are non-Newtonian in nature which do not have a direct relationship between stress and deformation rate, e.g., molten polymers, biological fluids, lubricants, mud and some fluids occurring naturally such as animal blood. These fluids have been distributed into different classes. Among these, one of the important models is Powell**–**Eyring fluid model which is many beneficial over the other non-Newtonian models, i.e., power law, Maxwell and micropolar fluid models. Although the considered model is very intricate and has favorite over the other fluid models, power law model is induced rather than the empirical relation from kinetic theory of liquid. It condenses to Newtonian relevance at high and low shear rates. Recently, the Powell–Eyring fluid was numerically studied by Akbar et al. [1]. They revealed the impact on Powell–Eyring due to magnetic field on Powell–Eyring fluid over a stretching surface. They analyzed that the velocity profile fell down with intensity enhancement of the magnetic flux and Powell–Eyring fluid parameters. Malik et al. [2] analyzed the flow of Powell–Eyring fluid above a stretching cylinder and considered two models, namely Reynaldo’s and Vogel’s models. They observed that boundary layer reduced for large Prandtl number values. Also, they analyzed that velocity profile increased by enhancing the values of suction parameter. The temperature profile reduces for large values of suction parameter. Ara et al. [3] illustrated the flow of Powell–Eyring fluid exponentially over a shrinking surface. They examined that velocity profile increased with the mass suction increment, while temperature profile showed opposite behavior. Also, the boundary layer thickness reduced with increase in Prandtl number. Nadeem and Saleem [4] observed the free and force convection flow by a stretching cone in the existence of mass and heat transfer. They observed that tangential velocity has opposite behavior for flow parameters. Moreover, they observed that skin friction coefficient increased due to the increment in ratio of buoyancy forces to flow parameter. Hayat et al. [5] examined the comparison of series and numerical solutions for flow of Powell–Eyring fluid with Newtonian heating. Also, they considered the internal heat generation and absorption. Malik et al. [6] used Keller box method and analyzed the MHD flow of tangent hyperbolic fluid over a stretching cylinder. Also in Refs. [7–10], authors investigated the Powell–Eyring fluid model in different physical conditions.

The heterogeneous and homogenous reactions are natural processes of chemical reactions. Several reactions progress very slowly, without a catalyst. The natural processes of heterogeneous and homogenous reactions are actually very complex including the consumption and production of reactant species at various rates on both the catalyst surface and fluid, i.e., reactions occurring in biochemical system, combustion and catalysis. Merkin [11] investigated the homogeneous and heterogeneous reactions for isothermal boundary layer flow model. Sarif et al. [12] have reported numerically the behavior of heat transfer past over a stretching plate. They demonstrated that boundary layer thickness varied with Prandtl number. Also, they investigated that temperature profile reduced by enhancement of Prandtl number and conjugate parameter. Kameswaran et al. [13] described the disintegration of the boundary layer flow past over a stretching surface with homogenous and heterogeneous reactions. They analyzed that velocity profile reduced with enhancement of concentration profile. Also, they observed that skin friction coefficient enhanced with increase in porous permeability parameter. They found that concentration reduced with the strength of heterogeneous reactions. Shaw et al. [14] examined the homogenous and heterogeneous reactions on the micropolar fluid flow and assumed porous stretching/shrinking sheet. They analyzed that momentum boundary layer thickness enhanced in the case of stretching sheet and fell down in the case of shrinking surface. Also, concentration profile has same results in the case of stretching/shrinking surface. They illustrated that the concentration reduced with the strength of the homogenous and heterogeneous reactions. The concentration of the reaction and fluid velocity on the surface increased with increase in stretching/shrinking parameters, while velocity enhanced with enhancement of micropolar parameter. The strength of homogenous and heterogeneous reactions reduces the strength of concentration reactants. Abbas et al. [15] observed the impacts of homogenous and heterogeneous reactions with stagnation point over a porous shrinking/stretching surface in attendance of transverse magnetic field. They observed that concentration of boundary layer thickness reduced by enhancing the suction and magnetic parameters in the case of stretching plate, but revealed increasing behavior for shrinking sheet case. Ellahi et al. [16] examined the characteristic effects of mixed convection MHD nanofluid over a vertical stretching surface. They noticed that velocity enhanced by enhancing the chemical dimensions and the radius of the gyration. References [17, 18] elaborate the recent work related to homogenous and heterogeneous reactions.

 Flow generated by a shrinking/stretching surface produces a great interest due to several applications in industries and engineering, e.g., two-dimensional steady flow is used for contracting and expanding the surface such as stretching/shrinking wrapping bundle, aluminum bottle industrial procedures, wrapping and hot rolling. These flows with heat transfer have a great importance in paper production, enhanced petroleum resources, food processing, etc. Nadeem et al. [19, 20] analyzed the boundary layer flow with point of stagnation. They observed that velocity field enhanced with enhancement of local injection and suction parameter. Akbar et al. [21] numerically analyzed the boundary layer flow of hyperbolic tangent fluid over a stretching surface. They analyzed that velocity profile reduced with increasing Hartmann number, while similar phenomena were noticed for velocity profile in power law index. They observed that Weissenberg number reduced the velocity profile. Also, they observed that increasing the Weissenberg number and power law increased the skin friction coefficient. Malik et al. [22] reported the flow of boundary layer over exponentially stretching cylinder. They illustrated that by enhancing the values of Casson fluid parameter velocity profile fell down while velocity profile increased by enhancing the mixed convection parameter. Also, they discussed that temperature profile fell down by enhancing the Reynolds number and Prandtl number. Tain et al. [23] observed the transfer of heat and fluid flow over a stretching plate with MHD and temperature-dependent viscosity. They analyzed that enhancement of Prandtl number decreases thermal boundary layer thickness. Lee [24] studied the boundary layer from smooth to rough surface with a step change. Zeeshan et al. [25] studied the effects of magnetic dipole on viscous ferrofluid past over a stretching surface with thermal radiation. Recent work on boundary layer flow and transfer of heat is mentioned in Refs. [18, 26–31].

The main motivations of this analysis are to analyze the effects of MHD flow of electrically conducting Powell–Eyring fluid over a stretching plate and also consider Newtonian heating with heterogeneous and homogenous reactions. The nonlinear expression concentration is transformed into ordinary differential expressions, and then, shooting method is employed in computing the result in MATLAB package. Numerical results are explained with tables and graphs.

## Method of solution

Consider incompressible two-dimensional steady flow of non-Newtonian fluid over a stretching surface with homogenous and heterogeneous reactions. The sheet is suddenly stretched such that the fluid flow with velocity *u*_*w*_ = *cx*, where *c* is a nonnegative constant. Consider two-dimensional coordinates system such that *x* is taken along with the plate, while the *y*-axis is taken at right angle to the sheet. We assume interaction between homogenous and heterogeneous reaction for a small model. Mathematical form of these reactions is given as:

1 $$A + 2B \to 3B,\;{\text{rate}} = K_{c} ab^{2} ,$$

2 $$A \to B,\;{\text{rate}} = K_{s} a.$$

In the above equations, *b* and *a* are the concentration of chemical species *B* and *A* and *Ki* (*i* = *s*, *c*) are the rate constant. The stream function *ψ* such that $u = \tfrac{\partial \varPsi }{\partial y}$ and $v = - \tfrac{\partial \varPsi }{\partial y}$ identically satisfies the continuity equation. All the governing laws are mathematically given as:

3 $$\frac{\partial v}{\partial y} + \frac{\partial u}{\partial x} = 0,$$

4 $$\begin{aligned} u\frac{\partial u}{\partial x} + v\frac{\partial u}{\partial y} & = \left( {\nu + \frac{1}{\rho \beta c}} \right)\left( {\frac{{\partial^{2} u}}{{\partial x^{2} }} + \frac{{\partial^{2} u}}{{\partial y^{2} }}} \right) - \frac{1}{{3\rho \beta c^{3} }}\frac{\partial }{\partial x}\left\{ {2\left( {\frac{\partial v}{\partial y}} \right)^{2} + \left( {\frac{\partial u}{\partial y} + \frac{\partial v}{\partial x}} \right)^{2} + 2\left( {\frac{\partial u}{\partial x}} \right)^{2} } \right\}\frac{\partial u}{\partial x} \\ & \quad - \frac{1}{{6\rho \beta c^{3} }}\frac{\partial }{\partial y}\left\{ {2\left( {\frac{\partial v}{\partial y}} \right)^{2} + \left( {\frac{\partial u}{\partial y} + \frac{\partial v}{\partial x}} \right)^{2} + 2\left( {\frac{\partial u}{\partial x}} \right)^{2} } \right\}\left( {\frac{\partial u}{\partial y} + \frac{\partial v}{\partial x}} \right) - \frac{{\sigma B^{2} u}}{\rho }, \\ \end{aligned}$$

5 $$\begin{aligned} u\frac{\partial v}{\partial x} + v\frac{\partial v}{\partial y} & = \left( {\nu + \frac{1}{\rho \beta c}} \right)\left( {\frac{{\partial^{2} v}}{{\partial x^{2} }} + \frac{{\partial^{2} v}}{{\partial y^{2} }}} \right) - \frac{1}{{6\rho \beta c^{3} }}\frac{\partial }{\partial x}\left\{ {2\left( {\frac{\partial v}{\partial y}} \right)^{2} + \left( {\frac{\partial u}{\partial y} + \frac{\partial v}{\partial x}} \right)^{2} + 2\left( {\frac{\partial u}{\partial x}} \right)^{2} } \right\}\left( {\frac{\partial u}{\partial y} + \frac{\partial v}{\partial x}} \right) \\ & \quad - \frac{1}{{3\rho \beta c^{3} }}\frac{\partial }{\partial y}\left\{ {2\left( {\frac{\partial u}{\partial x}} \right)^{2} + \left( {\frac{\partial u}{\partial y} + \frac{\partial v}{\partial x}} \right)^{2} + 2\left( {\frac{\partial v}{\partial y}} \right)^{2} } \right\}\frac{\partial v}{\partial y}, \\ \end{aligned}$$

6 $$u\frac{\partial T}{\partial x} + v\frac{\partial T}{\partial y} = \alpha \left( {\frac{{\partial^{2} T}}{{\partial y^{2} }} + \frac{{\partial^{2} T}}{{\partial x^{2} }}} \right) + \tau \left\{ {D_{B} \left( {\frac{\partial C}{\partial x}\frac{\partial T}{\partial x} + \frac{\partial C}{\partial y}\frac{\partial T}{\partial y}} \right) + \left( {\frac{{D_{T} }}{{T_{\infty } }}} \right)\left[ {\left( {\frac{\partial T}{\partial x}} \right)^{2} + \left( {\frac{\partial T}{\partial y}} \right)^{2} } \right]} \right\},$$

7 $$u\frac{\partial a}{\partial x} + v\frac{\partial a}{\partial y} = D_{A} \frac{{\partial^{2} a}}{{\partial y^{2} }} - k_{c} ab^{2} ,$$

8 $$u\frac{\partial b}{\partial x} + v\frac{\partial b}{\partial y} = D_{B} \frac{{\partial^{2} b}}{{\partial y^{2} }} + k_{c} ab^{2} ,$$

and the associated boundary conditions are as follows:

9 $$\begin{aligned} & u = u_{w} (x) = cx,\;\frac{\partial T}{\partial y} = - h_{s} T,\;v = 0,\,{\text{ at }}y = 0, \\ & \;T \to T_{\infty } ,\;u \to 0,\;{\text{ as}}\;y \to \infty ,\, \\ & D_{B} \left( {\frac{\partial b}{\partial y}} \right)_{y = 0} = - k_{s} a(0),\;D_{A} \left( {\frac{\partial a}{\partial y}} \right)_{y = 0} = k_{s} a(0), \\ & u(\infty ) = u_{e} (x),\;a(\infty ) = a_{0} ,\;b(\infty ) = 0. \\ \end{aligned}$$

In the above equations, *v* and *u* are the components of velocity in the *y*- and *x*-directions, respectively. *μ* is the dynamic viscosity, $\nu = \tfrac{\mu }{\rho }$ is the kinematic viscosity, *ρ* is the density, *β* is the fluid parameter, *σ* is the electrical conductivity of the fluid, *H* is the strength of magnetic field, *α* is the thermal diffusivity, for Newtonian heating *h*_*s*_ is the heat transfer parameter and *τ* is the ratio of heat capacity and effective heat capacity of the fluid. After applying the boundary layer approximation in Eqs. (3)–(8), we get the following formulas:

10 $$u\frac{\partial u}{\partial x} + v\frac{\partial u}{\partial y} = \left( {\nu + \frac{1}{\rho \beta c}} \right)\left( {\frac{{\partial^{2} u}}{{\partial y^{2} }}} \right) - \frac{1}{{2\rho \beta c^{3} }}\left( {\frac{\partial u}{\partial y}} \right)^{2} \frac{{\partial^{2} u}}{{\partial y^{2} }} - \frac{{\sigma uB^{2} }}{\rho },$$

11 $$u\frac{\partial T}{\partial x} + v\frac{\partial T}{\partial y} = \alpha \left( {\frac{{\partial^{2} T}}{{\partial y^{2} }}} \right) + \tau \left\{ {D_{B} \left( {\frac{\partial T}{\partial x}\frac{\partial C}{\partial x}} \right) + \frac{{D_{T} }}{{T_{\infty } }}\left( {\frac{\partial T}{\partial x}} \right)^{2} } \right\},$$

12 $$u\frac{\partial a}{\partial x} + v\frac{\partial a}{\partial y} = D_{A} \frac{{\partial^{2} a}}{{\partial y^{2} }} - k_{c} ab^{2} ,$$

13 $$u\frac{\partial b}{\partial x} + v\frac{\partial b}{\partial y} = D_{B} \frac{{\partial^{2} b}}{{\partial y^{2} }} + k_{c} ab^{2} .$$

The similarity transformations are defined as:

14 $$\begin{aligned} & \varPsi = (c\upsilon )^{{\tfrac{1}{2}}} xf(\eta ),\;\eta = \left( {\frac{c}{\upsilon }} \right)^{{\tfrac{1}{2}}} y,\;u = cxf^{\prime } (\eta ),\;\theta (\eta ) = \frac{{T - T_{\infty } }}{{T_{w} - T_{\infty } }}, \\ & v = - \sqrt {c\nu } f(\eta ),\;g(\eta ) = \frac{a}{{a_{0} }},\;h(\eta ) = \frac{b}{{a_{0} }}, \\ \end{aligned}$$

where *Ψ* is the stream function, and by using these similarity transformations, Eqs. (10)–(13) become

15 $$(1 + M)f^{\prime \prime \prime } - f^{\prime 2} + ff^{\prime \prime } - MBf^{\prime \prime 2} f^{\prime \prime \prime } - Hf^{\prime } = 0,$$

16 $$\theta^{\prime \prime } + f\mathop {Pr}\limits \theta^{\prime } = 0,$$

17 $$\frac{1}{Sc}g^{\prime \prime } + fg^{\prime 2} = 0,$$

18 $$\frac{\delta }{Sc}h^{\prime \prime } + fh^{\prime 2} = 0,$$

transformed conditions are as follows:

19 $$\begin{aligned} & f(0) = 0,\;f^{\prime } (0) = 1,\;f^{\prime}(\infty ) \to 0,\;\theta (0) = \frac{{\theta^{\prime } (0)}}{\gamma } + 1,\;\theta (\infty ) \to 0, \\ & g^{\prime } (0) = K_{s} g(0),\;g(\infty ) = 1, \\ \end{aligned}$$

and suppose further that diffusion coefficient *D*_*B*_ and *D*_*A*_ are equal, i.e., *δ* = 1, and by using these assumptions, following relation is obtained:

20 $$g(\eta ) + h(\eta ) = 1,$$

and by using Eq. (20), Eqs. (17) and (18) become

21 $$\frac{1}{Sc}g^{\prime \prime } + fg^{\prime 2} = 0,$$

with associated conditions

22 $$g(\infty ) = 1\;g^{\prime } (0) = K_{s} g(0)$$

In the above mathematical equations, prime denotes the differentiation w.r.t. *η*. $Pr = \tfrac{\nu }{\alpha }$ denoted the Prandtl number, $H = \tfrac{{\sigma B^{2} }}{\rho }$ is the Hartmann number, $M = \tfrac{1}{\mu \beta c}$ and $B = \tfrac{{a^{3} x^{2} }}{{2C^{2} \nu }}$ are fluid parameters, $K = \tfrac{{k_{c} a_{0}^{2} }}{c}$ is the measure of the strength of the homogenous reaction, $Sc = \tfrac{\nu }{{D_{A} }}$ is the Schmidt number, $\delta = \left( {\tfrac{{D_{B} }}{{D_{A} }}} \right)$ is the ratio of the coefficient of diffusion, $\gamma = \tfrac{{h_{s} }}{{\left( {\tfrac{a}{\nu }} \right)^{{\tfrac{1}{2}}} }}$ is the conjugate parameter for Newtonian heating, *T*_∞_ is the free stream and *T* is the fluid temperature, respectively. The quantity of practical interest such as skin friction coefficient is defined as:

23 $$C_{f} = \frac{{\tau_{w} }}{{\tfrac{1}{2}\rho u^{2} }},\;Nu = \frac{{xq_{w} }}{{k\left( {T_{w} - T_{\infty } } \right)}},$$

where

24 $$\begin{aligned} & \tau_{w} = \left[ {\left( {1 + \frac{1}{\beta c}} \right)\frac{du}{dy} - \frac{1}{{6\beta c^{3} }}\left( {\frac{\partial u}{\partial y}} \right)^{3} } \right], \\ & {\text{and}} \\ & q_{w} = - K\frac{\partial T}{\partial y}|_{y = 0} , \\ \end{aligned}$$

and the coefficient of skin friction and heat transfer coefficient become:

25 $$C_{f} Rex^{{\tfrac{1}{2}}} = \left[ {(1 + M)f^{\prime \prime } (0) - \frac{\lambda }{3}f^{\prime \prime 3} (0)} \right]\;NuRex^{{\tfrac{1}{2}}} = - \theta^{\prime } (0).$$

### Numerical solution

Equations (15)–(18) along with the associated boundary condition (19) are solved numerically by using shooting method. First we convert this system of equations into the system of first-order initial value problem and then solve this problem by using shooting method. Suppose

26 $$f = h_{1} ,\;f^{\prime } = h_{2} ,\;f^{\prime \prime } = h_{3} ,\;\theta = h_{4} ,\;\theta^{\prime } = h_{5} ,\;g = h_{6} ,\;g^{\prime } = h_{7} .$$

We get the system of ODEs

27 $$h_{1}^{\prime } = h_{2} ,$$

28 $$h_{2}^{\prime } = h_{3} ,$$

29 $$h_{3}^{\prime } = \frac{1}{{1 + M - MBh_{3}^{2} }}\left[ {h_{2}^{2} - h_{1} h_{3} + Hh_{2} } \right],$$

30 $$h_{4}^{\prime } = h_{5} ,$$

31 $$h_{5}^{\prime } = - \mathop {Pr}\limits \left( {h_{1} h_{5} } \right),$$

32 $$h_{6}^{\prime } = h_{7} ,$$

33 $$h_{7}^{\prime } = Sc\left[ {Kh_{6} \left( {1 - h_{6} } \right)^{2} - h_{1} h_{7} } \right],$$

with transformed initial conditions

34 $$\begin{aligned} & h_{1} (0) = 0,\;h_{2} (0) = 1,\;h_{2} (\infty ) = 0,\;h_{4} (0) = 1,\;h_{4} (\infty ) = 0, \\ & h_{6} (0) = 0,\;h_{6} (\infty ) = 0,\;h_{7} (0) = K_{s} h_{6} (0). \\ \end{aligned}$$

Choose a suitable initial approximations for *h*_3_ (0), *h*_5_ (0) and *h*_7_ (0) as 1, −1 and 1, respectively. Then, the system of first-order ODEs is solved using Runge–Kutta fifth-order technique. The convergence criterion for shooting method is 10^−6^ which absolute value of given and computed values of *h*_2_, *h*_4_ and *h*_6_. Thus, if the error tolerance, i.e., 10^−6^, is less than boundary residuals, then re-adjust initial guesses by Newton’s method. This loop is recycled again until the required criteria are satisfied.

## Results and discussion

**Fig. 1:**
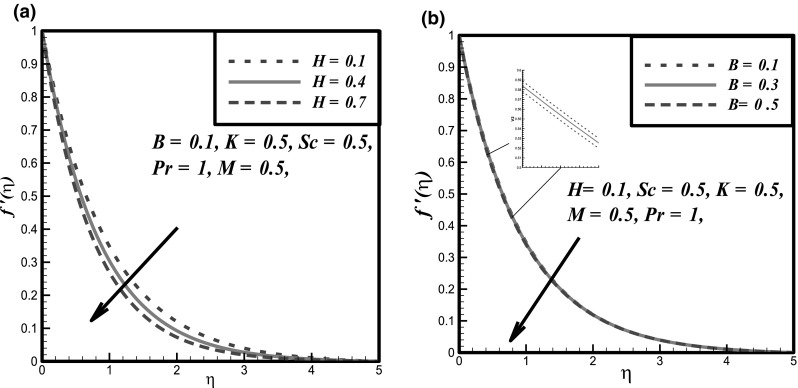
Fluid velocity curves against **a** *H* and **b** fluctuations in *B*

**Fig. 2:**
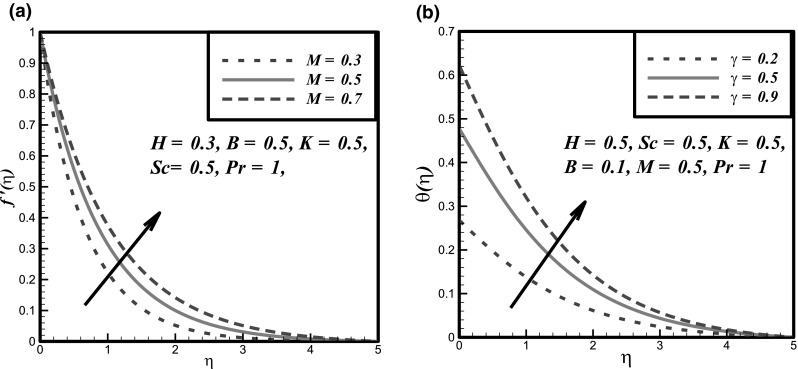
**a** Fluid velocity curves against fluctuations in *M*, **b** Effect of *γ* on temperature profile

**Fig. 3:**
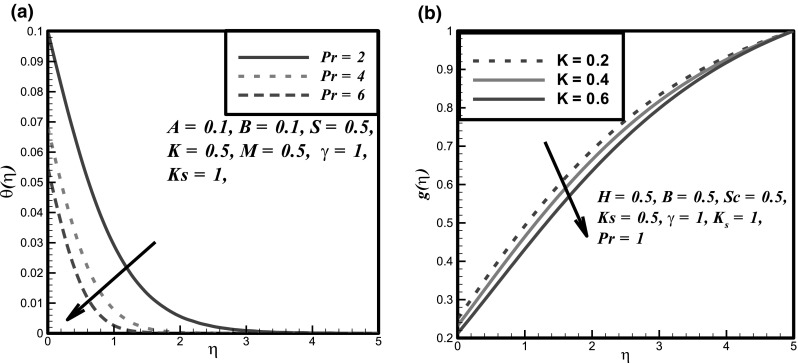
Effect of **a** *Pr* on temperature profile and **b** *K* on concentration profile

**Fig. 4:**
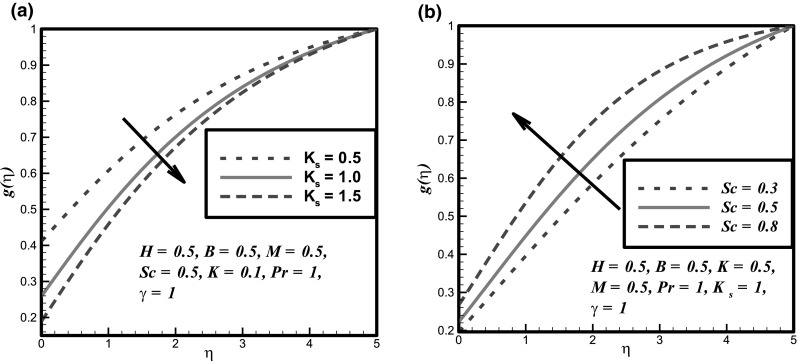
Impact of **a** *K*_*s*_ and **b** *Sc* on concentration profile

**Fig. 5:**
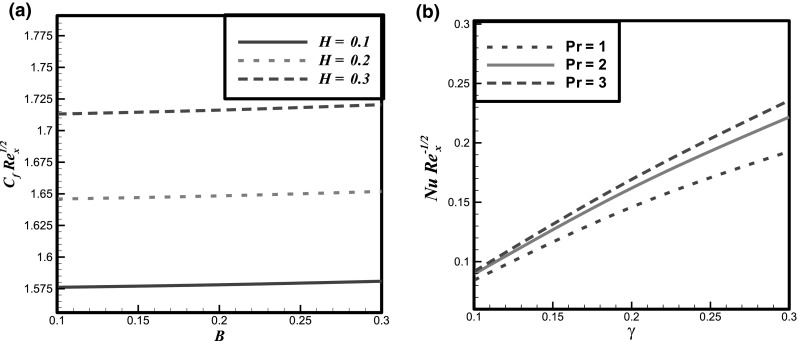
**a** Effect of *H* and *B* on skin friction coefficient, **b** effect of *Pr* and *γ* on Nusselt number

The impact of certain flow parameters on concentration, temperature and velocity profiles is visualized in this section. Figure 1a shows the impact of Hartmann number *H* on velocity profile. It is examined that by enhancing the Hartmann number *H* the velocity profile decreases. This is due to the retarding nature of the Lorentz forces which reduces the motion of the fluid in the boundary layer and enhances its temperature. Figure 1b shows the impact of fluid parameter *M* on velocity profile. It is evident that by hiking the values of fluid parameter *M* the velocity profile enhances. Because, it is illustrated that by enhancing the *M* the density of the fluid particles reduces which causes increase in velocity. Figure 2a presents the impact of fluid parameter *B* on velocity. From this figure, decrease in velocity profile is noticed for increase values of fluid parameter *B*. Because, for increment in fluid parameter *B*, the viscosity of fluid particle enhances, and due to this reason, the velocity profile reduces. Figure 2b shows the impact of Newtonian heating parameter *γ* on dimensionless temperature profile. As for Newtonian heating the value of conjugate parameter *γ* increase the thermal boundary layer thickness reduces eventually the temperature profile rise up. Figure 3a depicts the effect of Prandtl number *Pr* on dimensionless temperature profile. This figure reveals the reverse result for thermal boundary layer with increase in Prandtl number *Pr* when the thermal boundary layer falls down. The fluid has high conductivity for insignificant values of Prandtl number *Pr*. So by increasing the values of Prandtl number *Pr*, the thermal diffusivity reduces, which causes the decrease in temperature and thermal boundary layer of the fluid. Figures 3b and 4a show the effect of homogenous and heterogeneous reactions parameter *K* and *K*_*s*_ on concentration profile. It is clear from this figure that thickness of concentration boundary layer enhances with *η* and after certain values of *η* it has no effect of concentration profile in both cases. Figure 4b represents the impacts of Schmidt number *Sc* on mass transfer rate. As the Schmidt number *Sc* is the diffusivity ratio, i.e., mass and momentum diffusivity, the mass diffusivity is small for large number of Schmidt number *Sc* due to this fact that the concentration profile enhances. Figure 5a shows the behavior of skin friction coefficient versus fluid parameter *B* and strength of magnetic parameter *H*. It is manifested from figure that skin friction enhances by increasing the fluid parameter *B* and Hartmann number *H*. Figure 5b shows the effect of Prandtl number *Pr* and *γ* on Nusselt number. It is analyzed that for Newtonian heating the conjugate parameter *γ* reduces but Prandtl number *Pr* increase the Nusselt number. Table 1 shows the result of different parameters in skin friction coefficient. It is found that by increasing the fluid parameter *B* and Hartmann number *H* skin friction increases, while it shows opposite behavior in the case of fluid parameter *M*. Table 2 describes the significance of different parameters on Sherwood number and Nusselt number. These parameters show increasing behavior in mass transfer coefficient for large digit of Prandtl number *Pr* and conjugate parameter of Newtonian heating *γ*. Concentration profile increases for large values of Schmidt number *Sc* and falls down for homogenous reaction parameter *K*. Tables 3 and 4 show the excellent correlation for the numerical values of *θ* ^′^(0) and *f* ^′′^(0) with previous results of the literature against variation of Hartmann number *H* and Prandtl number *Pr*.

**Table 1:** Coefficient of skin friction against fluctuations in Hartmann number *H* and fluid parameters *B* and *M*

*H*	*B*	*M*	$C_{f} Re_{x}^{{\frac{1}{2}}}$
0.1	0.1	0.5	1.5761
0.2			1.6460
0.3			1.7131
0.1	0.1		1.5761
	0.2		1.5781
	0.3		1.5809
	0.5	0.5	1.5896
		0.6	1.5323
		0.7	1.4997

**Table 2:** Fluctuations in Nusselt number and Sherwood number against the parameters *Pr*, *γ*, *Sc* and *K*

*Pr*	*γ*	$NuRe_{x}^{{ - \frac{1}{2}}}$
1	0.1	0.0844
3		0.0895
2		0.1692
1	0.1	0.0844
	0.2	0.1459
	0.3	0.1928

*Sc*	*K*	$ShRe_{x}^{{ - \frac{1}{2}}}$
0.4	0.3	0.2250
0.6		0.2576
0.8		0.2900
0.4	0.3	0.2250
	0.4	0.2169
	0.5	0.2086

**Table 3:** Comparison of the present work for *f*″(0) with Hartmann number *H* when *Sc* = *Pr* = *K* = *B* = *0* and *M* = 0.0001

*H*	Fang et al. [29]	Akbar et al. [1]	Salahuddin et al. [30]	This study
0		1	1	1
0.5		−1.11803	−1.11801	−1.11803
1		−1.41421	−1.41418	−1.41423
5		−2.44949	−2.44942	−2.44942
10		−3.31663	−3.31656	−3.31658
100	−1.1180	−10.04988	−10.04981	−10.04987
500		−22.38303	−22.38293	−22.38294
1000		−31.63859	−31.63846	−31.63846

**Table 4:** Comparison of the present work for *θ*′(0) with *Pr* when *Sc* = *K* = *Pr* = 0 and *M* = 0.0001

*Pr*	Wang [31]	Mabood et al. [31]	Salahuddin et al. [30]	This study
0.07	0.0656	0.0655	0.0654	0.0654
0.20	0.1691	0.1691	0.1688	0.1689
0.70	0.4539	0.4539	0.4534	0.4539
2.00	0.9114	0.9114	0.9108	0.9113
7.00	1.8954	1.8954	1.8944	1.8944
20.00	3.3539	3.3539	3.3522	3.3532
70.00	6.4622	6.4622	6.4619	6.4619

### Concluding remarks

The study of two-dimensional homogenous and heterogeneous reactions in Powell–Eyring fluid model is considered numerically under the influence of MHD and Newtonian heating. The main results of this problem are:

1. A substantial reduction in the fluid parameters *B* and Hartmann number *H* was noticed in velocity profile.
2. A qualitatively opposite behavior was analyzed in the velocity profile for increasing values of for Newtonian heating parameter *γ* and Prandtl number *Pr*.
3. Concentration profile reduced by increasing homogenous and heterogeneous reactions parameter *K* and *K*_*s*_, but showed opposite behavior for Schmidt number *Sc*.
4. Skin friction coefficient enhanced when Hartmann number *H* and fluids parameters *B* and *M* increased.
5. Concentration profile enhanced for large values of homogenous reaction parameter *K*, heterogeneous reaction parameter *K*_*s*_ and Schmidt number *Sc*.
